# Mechanic’s hand; is it a prodromic sign of disease relapse of anti-synthetase syndrome; a case report

**DOI:** 10.1186/s41927-021-00195-2

**Published:** 2021-07-26

**Authors:** C. T. Rosa, A. S. Thilakarathne, L. A. Senevirathne, A. Wijeyeratna, D. Munidasa

**Affiliations:** Rheumatology and Rehabilitation Hospital, Ragama, Sri Lanka

**Keywords:** Mechanic’s hands, Anti-synthetase syndrome, Myositis, Cyclophosphamide, Interstitial lung disease, Case report

## Abstract

**Background:**

Anti-synthetase syndrome is the collection of myositis and/or interstitial lung disease with the presence of various antibodies directed against an aminoacyl transfer RNA synthetase. Anti Jo − 1 antibody is the commonest of these antibodies and its presence is characteristically associated with the dermatological manifestation of mechanic’s hands. However, in the absence of other features, whether the presence of mechanic’s hands could be considered as a prodromic sign of disease relapse is not proven. We would like to present a patient who developed mechanic’s hands and subsequently went on to have recurrence in her myositis.

**Case presentation:**

A 45-year-old female initially presented with a progressive proximal muscle weakness. Her muscle enzymes were elevated, EMG and biopsy were also in keeping with an inflammatory myositis. Subsequently she was found to have an interstitial lung disease with a non-specific interstitial pneumonitis pattern radiologically. Her anti Jo-1 was positive. However, she did not have any dermatological manifestations at the time. With immunosuppressive therapy she achieved remission which lasted for about 2 years. Then she developed fissuring and cracking of the palms and fingers suggestive of mechanic’s hands without any muscle pain, weakness and elevation of muscle enzymes. A few months later she did develop muscle pain, weakness and elevation of muscle enzymes heralding a disease relapse.

**Conclusion:**

The presence of mechanic’s hands without other features should be considered as a prodromic sign of disease relapse.

## Background

Anti-synthetase syndrome is generally considered as autoimmune condition characterized by inflammatory myositis, interstitial lung disease with the presence of antibodies directed against an aminoacyl transfer RNA synthetase. The commonly seen antibody among these group of patients is the anti Jo-1 antibody which is associated with the presence of mechanic’s hands. Mechanic’s hands are described as fissuring and cracking of the palms, radial side of the distal end of fingers and ulnar aspect of the thumb. While mechanic’s hands are well described in the medical literature whether its appearance is a prodromic sign of disease relapse is less known. We would like to present a patient with Anti Jo-1 positive anti-synthetase syndrome who developed mechanic’s hands, few months prior to systemic relapse.

## Case presentation

A 45-year-old South Asian woman presented with a progressively worsening of proximal muscle weakness. Initially there was a mild pain in her thighs and arms which was followed by a difficulty getting up from the seated position and inability of raising her arms. There was no dysphagia, regurgitation or difficulty in breathing. During this particular presentation there was no dermatological manifestations suggestive of dermatomyositis or mechanic’s hands. There was no history suggestive of Raynaud’s phenomenon or any joint pains. There was a mild nonproductive cough which developed a few weeks after the onset of the muscle symptoms without any notable shortness of breath of exertion or wheezing. There was no other evidence of any connective tissue diseases.

There was no ptosis, double vision or fatiguability to suggestive myasthenia. Patient appeared to be clinically euthyroid without any overt features of hypothyroidism. There was no history of statin use at the time of the presentation or prior to that. Patient denied any similar history previously nor was there a history of a similar disease among her family members.

Examination revealed bilateral symmetrical proximal muscle weakness with a muscle power of 4/5 in all four limbs. Reflexes were intact without any muscle wasting. There were no overt features of dermatomyositis. Auscultation of the lungs revealed a few bilateral basal fine end inspiratory crepitations suggestive an interstitial lung disease. Second heart sound was of normal intensity. A diagnosis of inflammatory myositis was considered with a concurrent interstitial lung disease. With this clinical text she was thoroughly investigated.

**Fig. 1:**
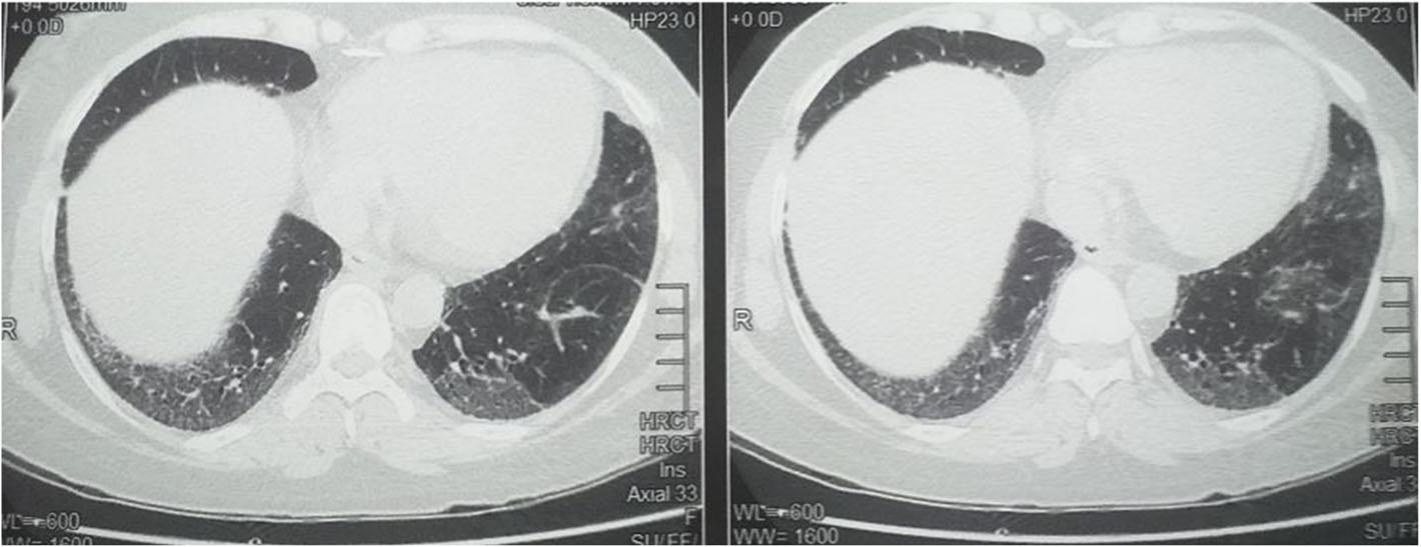
High resolution CT scan showing bilateral basal ground glass opacification suggestive of non-specific interstitial pneumonitis

Creatinine phosphokinase levels were elevated with a value of 1435 u/l. Inflammatory markers were elevated with a ESR of 54 though the CRP was only slightly elevated. ANA was negative. Findings on electromyography was consistent with an inflammatory myositis. Muscle biopsy which was carried out revealed inflammatory cells in between the muscle fibers causing complete necrosis of some of the muscle fibers. Anti Jo-1 antibody was positive. HRCT revealed bilateral homogenous sub pleural basal ground glass opacification suggestive of non-specific interstitial pneumonitis (Fig. 1). She was started on oral prednisolone 1 mg/kg/day with azathioprine 50 mg per day which was later increased to 100 mg per day. Subsequently her muscle power improved, CPK levels normalized and the cough which was present improved.

She attended her follow up clinic visits and was compliant with her medication. She was in remission for 2 years with low dose prednisolone and azathioprine. She was asymptomatic with no muscle pains, proximal muscle weakness and CPK levels were within normal range. She continued to be free from any cough or shortness of breath. ESR was persistently normal with values in the mid 20’s.

**Fig. 2:**
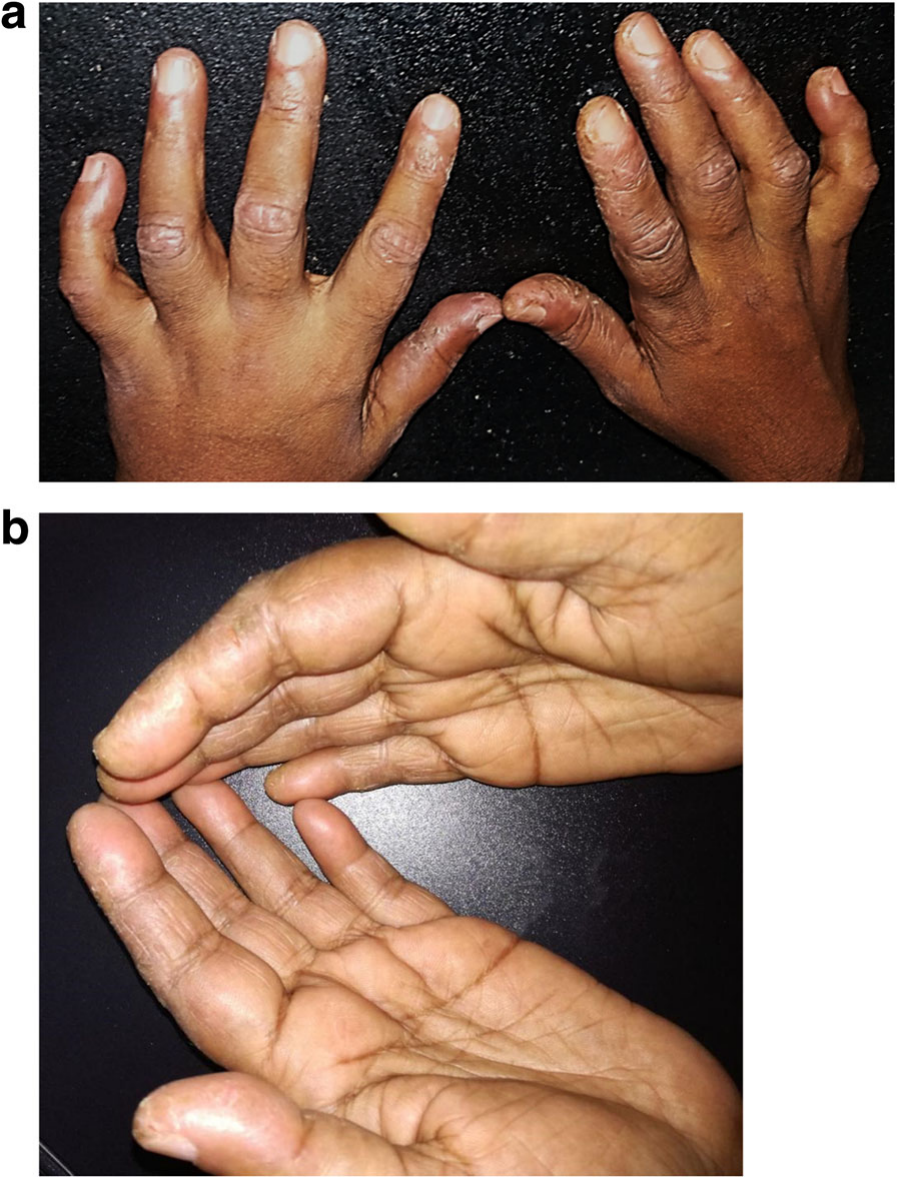
**a** Fissuring and cracking of hands suggestive of mechanic’s hands at the onset of disease relapse. **b** Fissuring and cracking of hands suggestive of mechanic’s hands at the onset of disease relapse

**Fig. 3:**
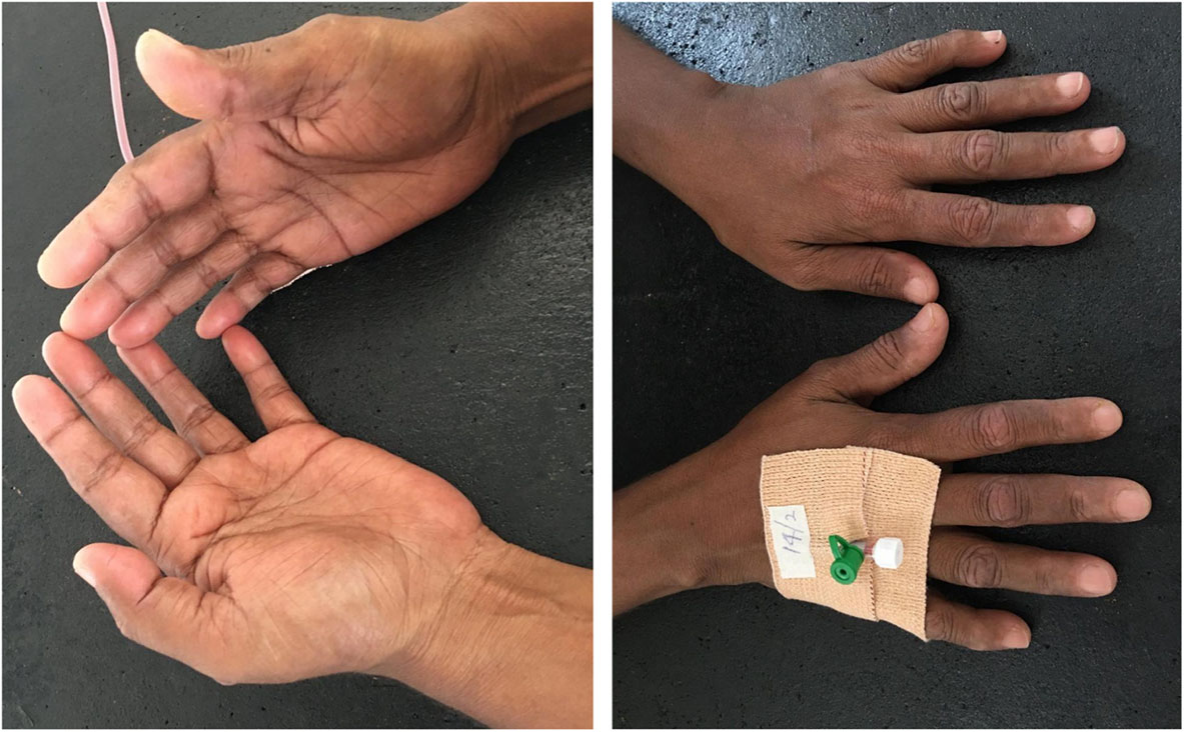
Resolution of mechanic’s hands after IV cyclophosphamide

Following a period of unsatisfactory compliance of 4 weeks, she presented with fissuring and cracking of the fingers suggestive of mechanic’s hands (Fig. 2) without any muscle pain or weakness. She denied any recurrence of the cough which she had during the initial disease presentation and did not have any shortness of breath. Her CPK was within normal limits at that time. Though there was no evidence of a systemic relapse, the steroid dose was up titrated while continuing azathioprine. However, the mechanic’s hands did not improve and subsequently in the following month she started to complain of a pain in her thighs and arms with a subtle weakness. Her CPK had risen to 1427 u/L and ESR risen to 47. Given the overall clinical picture it was decided that she required escalation of her immunosuppressive therapy. She was started on IV cyclophosphamide 500 mg every 2 weekly and after 3 doses the skin manifestations started to resolve and CPK had normalized (Fig. 3). Hydroxychloroquine 200 mg daily was added as patient had prominent dermatological manifestations with photosensitivity (Table 1).

Repeat HRCT revealed stable nonspecific interstitial pneumoniae without any radiological progression. Lung function tests revealed a restrictive lung disease with a FVC of 78% of predicted, FEV1 of 94% of predicted with FEV1/FVC of 124%. DLCO was 49%.

**Table 1:** timeline of events

Event	Timeline
Development of proximal muscle weakness (1st episode)	January 2017
Diagnosis and Immunosuppressive therapy with oral steroids and azathioprine	February 2017
Clinical Remission	March 2017 – September 2019
Development of Mechanic’s hands	October 2019
Development of proximal muscle pain, weakness and elevation of CPK (2nd episode)	December 2019
Escalation of immunosuppressive therapy with cyclophosphamide	December 2019 – February 2020
Resolution of mechanic’s hands and normalization of CPK, pain and weakness	January 2020

## Discussion and conclusion

Anti-synthetase syndrome is the presence of inflammatory myositis and/or interstitial lung disease with the presence of antibodies directed against an aminoacyl transfer RNA synthetase. Some cases may go on to develop arthritis, Raynaud’s phenomenon and mechanic’s hands. This patient during the initial presentation only had evidence of myositis and interstitial lung disease. However, prior to the subsequent relapse of the disease she developed mechanic’s hands. In fact, the mechanic’s hands preceded the muscle symptoms by a few months.

15–25% of patients with anti-synthetase syndrome will have anti Jo-1 antibody positivity while several other antibodies have been described with its unique clinical phenotypes. The anti Jo-1 positive anti synthetase syndrome is associated with myositis, interstitial lung disease, non-erosive arthritis, Raynaud’s phenomenon and mechanic’s hands.

Pathogenesis of mechanic’s hands in the setting of anti-synthetase syndrome is not clear. Histologically there is marked hyperkeratosis, focal parakeratosis, psoriasiform acanthosis, and colloid bodies in the epidermis. These are similar to histological features of psoriasis. Similarities in the histological changes and the successful use of ustekinumab in refractory mechanic’s hands could mean a pathogenesis similar to psoriasis [1, 2].

While mechanic’s hands are described as part of the clinical manifestations, whether its presence can be utilized as a prodromic sign of disease relapse needs further evaluation. In our case, the patient was in remission when she developed mechanic’s hands. Myositis was in remission evident by absence of muscle pains, proximal muscle weakness and by normalization of CPK and ESR. From a respiratory standpoint, she had a cough without any shortness of breath at the time of initial diagnosis however clinically improved with immunosuppressive therapy. Though spirometry results are not readily available, she remained clinically and radiologically stable respiratory wise throughout the disease course.

Few months developing mechanic’s hands she did go on to develop proximal muscle pain, weakness and CPK elevation. Subsequent escalation of immunosuppressive therapy led to the resolution of the mechanic’s hands and improvement in muscle power and normalization of CPK levels.

Therefore, the chronology of events in this case is suggestive of mechanic’s hands being considered as a prodromic sign of disease relapse.

In one previously reported case, the appearance of mechanic’s hands had preceded the development of myositis and arthritis [3]. In another case with anti Jo-1 positive anti-synthetase syndrome, mechanic’s hands preceded the development of respiratory manifestations [4]. However, in contrast there is another reported case of anti Jo − 1 antibody positive anti-synthetase syndrome with mechanic’s hands which was refractory to conventional treatment while other systemic manifestations were under control for a period of 3 years. Though cutaneous manifestations were resistant to therapy, the continuation of immunosuppressive therapy including MMF and methotrexate could have prevented a systemic disease relapse in this particular case [2].

Therefore, while further evidence is required before any solid conclusion can be made, our patient’s clinical scenario adds to the current evidence that mechanic’s hands should be considered as a prodromic sign of disease relapse.

## Data Availability

Not applicable.

## Abbreviations

RNA: Ribonucleic acid
CPK: Creatinine phosphokinase
HRCT: High resolution computer tomography
FVC: Forced vital capacity
FEV1: Forced expiratory volume
DLCO: Diffusion capacity of lungs for carbon monoxide
